# Delayed Postoperative Hyponatremia Following Endoscopic Transsphenoidal Surgery for Non-Adenomatous Parasellar Tumors

**DOI:** 10.3390/cancers12123849

**Published:** 2020-12-20

**Authors:** Hirotaka Hasegawa, Masahiro Shin, Noriko Makita, Yuki Shinya, Kenji Kondo, Nobuhito Saito

**Affiliations:** 1 Department of Neurosurgery, The University of Tokyo Hospital, Tokyo 113-8655, Japan; hirohasegawa-tky@umin.ac.jp (H.H.); yukishinya6155@gmail.com (Y.S.); nsaito-tky@umin.net (N.S.); 2 Department of Neurologic Surgery, Mayo Clinic, Rochester, MN 55905, USA; 3 Department of Endocrinology and Nephrology, The University of Tokyo Hospital, Tokyo 113-8655, Japan; norimaki-tky@umin.ac.jp; 4 Department of Otolaryngology, The University of Tokyo Hospital, Tokyo 113-8655, Japan; kondok-tky@umin.ac.jp

**Keywords:** cerebral salt-wasting syndrome, endoscopic transnasal approach, hyponatremia, postoperative complication, skull base tumor, syndrome of inappropriate antidiuretic hormone secretion

## Abstract

**Simple Summary:**

Delayed postoperative hyponatremia is a known complication after transsphenoidal surgery for pituitary adenoma, but this may occur after surgery for parasellar non-adenomatous skull base tumors (NASBTs). Due to their scarcity, however, little is known about this condition. Through a retrospective review of 30 patients with NASBTs and detailed analyses on perioperative serial sodium level, we found that serological hyponatremia (sodium ≤ 135 mmol/L) occurred in eight (27%) on postoperative day 7–12, with four (13%) of them being symptomatic. Four (50%) showed weight loss and hemoconcentration suggesting cerebral salt wasting type, and three (38%) showed weight gain and hemodilution, suggesting a syndrome of inappropriate antidiuretic hormone secretion. Hyponatremia should be recognized as a possible complication after transsphenoidal resection of NASBTs. Intraoperative extradural retraction of the pituitary gland was the only significant factor for serological hyponatremia, suggesting the maneuver and subsequent dysregulation of the hypothalamo-hypophyseal axis may be responsible.

**Abstract:**

Little is known about delayed postoperative hyponatremia (DPH) accompanied with transsphenoidal surgery for non-adenomatous skull base tumors (NASBTs). Consecutive data on 30 patients with parasellar NASBT was retrospectively reviewed with detailed analyses on perioperative serial sodium levels. Serological DPH (sodium ≤ 135 mmol/L) was observed in eight (27%), with four (13%) of them being symptomatic. DPH developed on postoperative day 7–12 where the mean sodium levels were 134 mmol/L (a mean of 7 mmol/L drop from the baseline) in asymptomatic and 125 mmol/L (a mean of 17.5 mmol/L drop from the baseline) in symptomatic DPH. Serological DPH was accompanied with “weight loss and hemoconcentration (cerebral salt wasting type)” in four (50%), “weight gain and hemodilution (syndrome of inappropriate antidiuretic hormone secretion type)” in three (38%), and no significant weight change in one. Intraoperative extradural retraction of the pituitary gland was the only significant factor for serological DPH (*p* = 0.035; odds ratio, 12.25 (95% confidence interval, 1.27–118.36)). DPH should be recognized as one of the significant postsurgical complications associated with TSS for NASBTs. Although the underlying mechanism is still controversial, intraoperative extradural compression of the pituitary gland and subsequent dysregulation of the hypothalamo-hypophyseal axis may be responsible.

## 1. Introduction

Transsphenoidal surgery (TSS) is a widely used approach for pituitary adenomas [1,2]. Although this approach works efficiently as a definitive therapeutic modality with minimal invasiveness, one of the significant complications is delayed postoperative hyponatremia (DPH). This complication arises in around 20% of patients in a delayed fashion with serum sodium reaching the lowest level around postoperative day (POD) 7 and causes headache, dizziness, nausea, vomiting and, most profoundly, altered mental status and seizures that are potentially catastrophic, representing the most common cause of 30-day unplanned readmission after TSS for pituitary adenoma in the United States [3,4,5,6,7,8,9,10].

In general, syndrome of inappropriate antidiuretic hormone (ADH) secretion (SIADH) and cerebral salt-wasting syndrome (CSWS) are two major causes of DPH [8,11,12,13,14,15]. The essential difference between those two conditions is volume status: SIADH involves hypervolemic hyponatremia, whereas CSWS demonstrates hypovolemic hyponatremia. In cases of DPH among patients with pituitary adenoma, mechanical stress to the pituitary system is hypothesized to induce an aberrant release of ADH from degenerated axon terminals of the magnocellular neurosecretory neurons, causing SIADH [5,16]. On the other hand, significant uncertainty remains regarding the pathophysiology of CSWS, but aberrant release of natriuretic peptides together with decreased sympathetic input to the kidney are believed to be involved [12,13,17,18,19].

Considering that mechanical stress to the pituitary system may play an important role for DPH following TSS for pituitary adenomas [5,16], DPH would theoretically develop after TSS for non-adenomatous skull base tumors (NASBTs) as well, as long as they are located in parasellar regions and potentially require mechanical stress on the pituitary system during resection. However, little is known about DPH among NASBTs. Given that a variety of midline and paramidline ventral skull base tumors that had previously been treated exclusively with invasive open skull base surgeries have recently become potential targets of TSS according to recent advances in endoscopic techniques [20,21,22,23,24,25,26,27,28], it is of scientific importance to clarify whether TSS for NASBTs is followed by DPH. We, therefore, conducted a comprehensive retrospective study to clarify the incidence, type, clinical course, and risk factors of DPH, as well as to estimate the underlying mechanisms.

## 2. Results

### 2.1. Baseline Characteristics

Detailed baseline characteristics, pathological diagnoses, and tumor locations are summarized in Table 1. During TSS, extradural retraction of the gland was needed in 15 (50%) and physical contact with the stalk in 5 (17%).

### 2.2. Symptomatic and Serological DPH

A serological DPH was observed in eight patients (27%) (Table 2). Among them, four patients (13%) became symptomatic (nausea and fatigue in all patients, emesis in one, and headache in one). Data on weight change were available in all eight patients with serological DPH and 17 patients without serological DPH. Among patients with serological DPH, four patients (50%) showed weight loss (mean, −5.3%; range, −6.9–−3.6%), three patients (38%) showed weight gain (mean, +5.2%; range, +2.4–+7.7%), and one patient (12%) showed <±1% weight change (−0.5% weight loss) at the time of the nadir. Patients who showed weight loss exhibited a mean +6.7% increase in hematocrit level; patients with weight gain displayed a mean −6.4% decrease in hematocrit level. One patient with indefinite weight change had a +26.7% increase in hematocrit level at the time of the nadir; this patient received blood transfusion for anemia on POD 1. Among patients without serological DPH, 14 patients (82%) showed weight loss (mean, −4.7%; range, −8.3–−1.6%), one patient (6.0%) showed +3.1% weight gain, and two patients (6.0%) showed <±1% weight change (−0.6% and −0.9% weight loss) in phase 3. Those who showed weight loss displayed an increase in hematocrit (mean, +8.1%) in phase 3.

**Figure 1:**
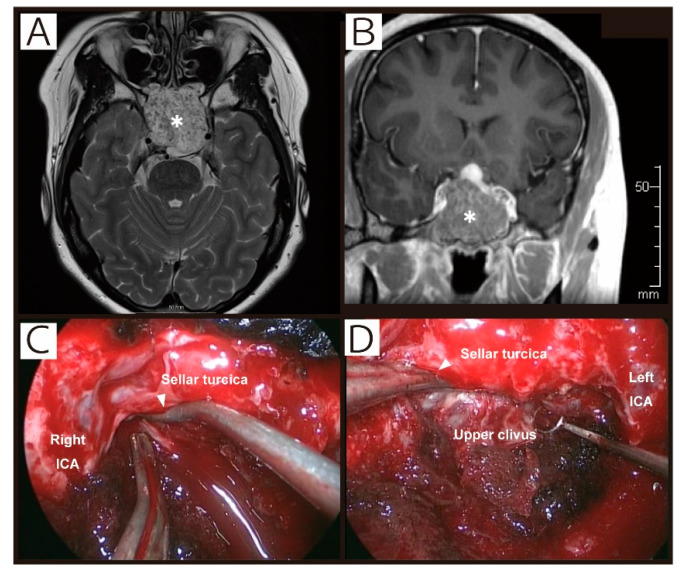
Patient #3. A 38-year-old woman presented with left visual impairment and abducens nerve palsy. MRI ((**A**), T2-weighted image; (**B**), contrast-enhanced T1-weighted image) demonstrated a large skull base mass extending to the clivus, sphenoid sinus, bilateral petrous apex and cavernous sinus. The pituitary gland was compressed upward, but the interface between the dura and tumor was preserved. During the extended transsphenoidal approach, tumors at the posterior cavernous sinus ((**C**), intraoperative image) and dorsum sellae ((**D**), intraoperative image) were exposed and removed with retraction of the dura of the pituitary gland upward by suction tube. White arrowheads show the point of extradural retraction. The pathological diagnosis was chordoma. The postoperative course was uncomplicated, but the patient developed nausea and severe fatigue, and turned out to have severe hyponatremia (serum sodium level, 126 mmol/L) on POD 7 with a weight gain pattern (2.4% increase from baseline). Sodium level improved to 132 mmol/L on POD 8 only with oral salt supplementation, and symptoms resolved accordingly. *, Tumor.

**Figure 2:**
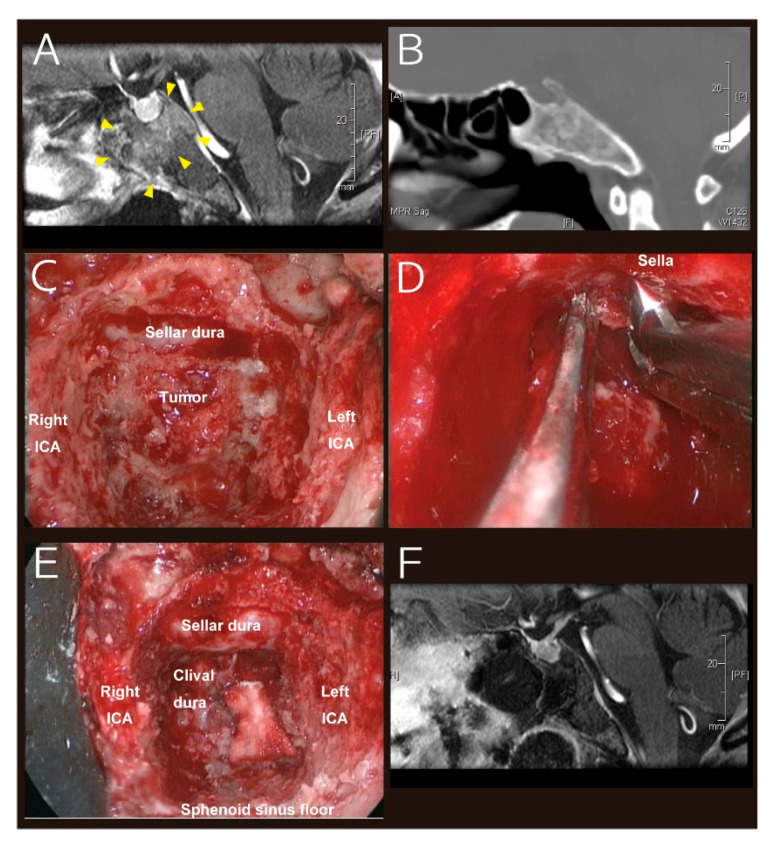
Patient #2. A 69-year-old woman presented with diplopia. MRI demonstrated an irregularly enhancing midline clival lesion ((**A**), contrast-enhanced T1-weighted image; yellow arrowheads indicate the mass). Of note, the patient showed a conchal-type sphenoid sinus ((**B**), computed tomography). An extended transsphenoidal transclival approach was selected. After opening the sphenoid sinus, plenty of clival bone had to be removed to expose the dura of the sellar floor and the tumor ((**C**), intraoperative image). Since the tumor was located under and behind the sella turcica, retraction of the sellar dura was necessary to remove the tumor ((**D**), intraoperative image). Gross total resection was achieved ((**E**), intraoperative image). MRI on POD 1 shows a good resection cavity with intact pituitary gland ((**F**), contrast-enhanced T1-weighted image). Pathological examination revealed chordoma. Sodium levels demonstrated a mild increase in phases 1 and 2, then dropped to 123 mmol/L on POD 10 when the patient developed severe fatigue. Sodium level improved to 134 mmol/L on POD 12 only with oral salt supplementation and mild fluid restriction, and the symptoms resolved accordingly.

All four symptomatic patients received salt supplementation with mild fluid restriction but one patient with a weight gain pattern developing moderate fatigue and nausea, resulting in rapid recovery within two days. Representative cases are illustrated in Figure 1 and Figure 2.

### 2.3. Detailed Course of Sodium Levels

**Figure 3:**
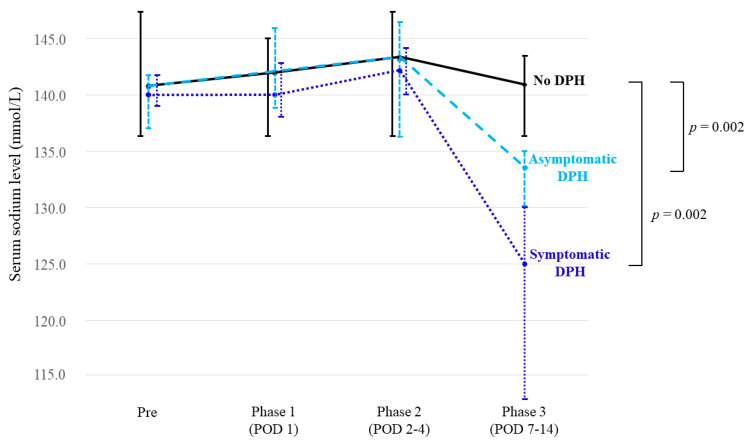
Line graph representing the courses of serum sodium level in patients with no (solid black line), asymptomatic (dashed light blue line), and symptomatic (dotted blue line) DPH. Vertical lines indicate standard deviations at each time point.

The detailed course of sodium levels is summarized in Table 3 and Figure 3. Median sodium levels did not differ significantly among patients without DPH, patients with asymptomatic DPH, and patients with symptomatic DPH, before surgery, in phase 1, and in phase 2. However, significant change was seen in phase 3, when median sodium level in patients without DPH remained stable (141 mmol/L); whereas levels in patients with asymptomatic DPH (134 mmol/L; *p* = 0.002) and symptomatic DPH (125 mmol/L; *p* = 0.002) were significantly lower than in patients without DPH.

Median changes in sodium level at nadir in phase 3 from baseline were 0.5 mmol/L (−3–+5 mmol/L) in patients with no DPH, −7 mmol/L (−3–−11 mmol/L) in asymptomatic DPH, and −17.5 mmol/L (−12–−27 mmol/L) in symptomatic DPH.

### 2.4. Risk Factors Associated with DPH

Results of risk factor analyses are summarized in Table 4. Extradural retraction of the pituitary gland was the only significant factor for serological DPH (*p* = 0.035; odds ratio, 12.25 (95% confidence interval, 1.27–118.36)). It also showed close correlation to symptomatic DPH without statistical significance (*p* = 0.0996; odds ratio, 12.1 (95% confidence interval, 0.6–248.5)). Among the 15 patients who underwent extradural retraction of the pituitary gland during surgery, symptomatic DPH developed in four (26.7%) and serological DPH in seven (46.7%). In contrast, among the 15 patients who did not require this manipulation, only one (6.7%) developed asymptomatic DPH.

### 2.5. Other Electrolytic and Endocrinological Abnormalities

Postoperatively, persistent diabetes insipidus requiring prolonged ADH supplementation was newly confirmed in 1 patient (3.3%) with posterior fossa neurenteric cyst who required manipulation by the stalk. Mild transient diabetes insipidus was confirmed in one patient (Patient #5) in phase 1 who eventually developed asymptomatic DPH (SIADH pattern) in phase 3. This patient also required direct manipulation of the pituitary stalk during surgery.

Hypopituitarism requiring prolonged cortisol supplementation occurred in two patients, including one with tuberculum sella meningioma and 1 with craniofacial meningioma extending to the sphenoid sinus and sellar region. Of these, one patient (Patient #4) developed symptomatic DPH on POD 7.

## 3. Discussion

In our series, serological DPH occurred in 27% of patients with NASBT, half of whom became symptomatic (13% of all patients). The course of DPH was largely benign and self-limited, and conservative management seemed to work well, but we should be aware that, in some cases, serum sodium levels may drop significantly and cause profound symptoms. DPH should be recognized as one of the significant postsurgical complications associated with TSS for NASBT.

In terms of volume status, roughly 70% of all patients demonstrated some extent of weight loss with hemoconcentration in phase 3. Since most patients with weight loss did not show dysnatremia, the weight loss itself may not necessarily be pathological; rather, this loss might be explained by various issues, including surgical stress, decreased oral intake, exhaustion, and inflammation. Notably, the impact of surgeries for NASBTs might be more significant than simple pituitary adenoma surgery. Hemoconcentration might be explained by cancellation of sodium and water retention due to transient use of hydrocortisone. Nevertheless, some patients clearly suffered DPH on top of weight loss, suggesting the presence of some underlying pathological mechanism resulting in a negative sodium-water balance, consistent with CSWS. On the other hand, approximately 40% of patients with DPH showed weight gain with hemodilution, consistent with SIADH. This weight gain pattern was rarely seen in patients without DPH and, thus, may directly suggest a pathological process.

Given that the hypothalamo-hypophyseal axis is a strong regulator of body fluid and sodium metabolism [29], dysregulation of this axis is the most likely underlying cause of DPH. In this axis, ADH produced in the hypothalamus and released from the neurohypophysis contributes to volume expansion and lowers the serum sodium level through water reabsorption in the renal collecting duct [29,30,31]. In a proposed theory of DPH associated with pituitary adenoma, the first step would be a temporary arrest of ADH secretion due to damages to the magnocellular osmoregulatory system in the pituitary, leading to a mild sodium increase or diabetes insipidus if severe. Second, ADH will be subsequently released from the degenerating magnocellular neurons after some days following surgery, causing water retention and a resultant decrease in sodium level [5,8]. Considering that there are some similarities between DPH associated with NASBT and pituitary adenoma, such as the timing of nadir (around POD 7), incidence (symptomatic, 13%; both symptomatic and asymptomatic; 27%), and self-limiting course [10,32], a similar pathophysiological mechanism may contribute to DPH associated with NASBTs. Of note, however, the fact that the CSWS pattern was seen more often than the SIADH pattern is somewhat in contrast with DPH associated with pituitary adenoma, where the majority reportedly show a SIADH pattern [6,8,9,10,33]. We have no good explanation for this difference. Given that atrial natriuretic peptide (ANP) is a potent diuretic and natriuretic hormone and locally produced in the hypothalamo-hypophyseal axis, it is likely associated with CSWS-type DPH. In a physiological condition, ANP causes decreases in volume and serum sodium level through substantial ANP release from the atrium into the systemic circulation, which might be mediated by ANP-dependent oxytocin release from the neurohypophysis [29,34,35,36,37,38]. Therefore, both these peptides would work toward a decrease in serum sodium level if aberrantly released; however, the functions of ADH and ANP/oxytocin are opposite in terms of body water metabolism, presumably resulting in SIADH and CSWS, respectively.

**Figure 4:**
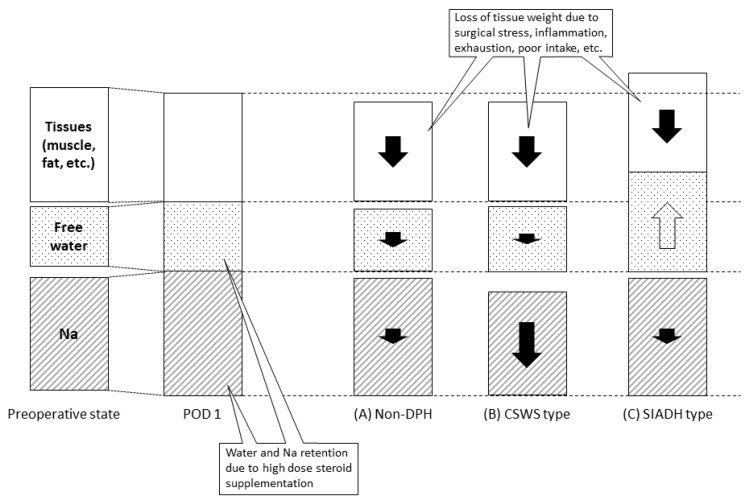
Schematic drawings representing changes in volume status in each condition. On POD 1, while a slight decrease in tissue weight occurs due to surgical stress, positive water and sodium balance is caused by high-dose steroid supplementation, resulting in stable body weight compared to the preoperative state. In the later postoperative period, further loss of tissue weight results as a consequence of a variety of surgery-related factors, including surgical stress, inflammation, exhaustion, poor intake, etc. On top of this weight loss, in patients without DPH, steroid tapering results in normalization of positive water and sodium balance, leading to weight loss with an increased hematocrit. Physiological hematopoiesis to make up for intraoperative blood loss may also explain the increase in hematocrit. In patients with CSWS-type DPH, absolute sodium loss due to dysregulation of the hypothalamo-hypophyseal axis and ADH secretion accompanied by salt losing may accelerate hyponatremia with hemoconcentration. In patients with SIADH-type DPH, positive water balance will cause weight gain and hemodilution.

The presence of various weight change patterns in our patients with DPH seems contradictory. However, this would be possible if mechanical stress to the neurohypophysis causes aberrant release of both ADH and ANP/oxytocin; either weight gain, weight loss, or indefinite weight change would depend on whether ADH or ANP/oxytocin function becomes predominant or remain relatively balanced. In our series, Patients #1, #3, and #5 showed DPH with a SIADH pattern, which may have resulted from predominant ADH function, and Patients #2, #6, #7, and #8 displayed DPH with a CSWS pattern, which may have resulted from a predominance of ANP/oxytocin function (Figure 4). In any case, intraoperative extradural compression of the pituitary gland seems to have been responsible for this dysregulation. Meticulous follow-up would thus be warranted for patients needing this manipulation.

Nevertheless, the true mechanisms underlying DPH remain complicated, and several other complex pathophysiological conditions may exist at the same time [8,10,33,39,40,41]. Direct dissection at the tumor-gland interface could theoretically represent a risk for DPH as well, as this procedure is commonly used in TSS for pituitary adenoma and is presumably responsible for mechanical stress to the pituitary gland. However, since most NASBTs were extradural lesions, only one case in this study required such direct dissection and, thus, we did not test the impact of this procedure. Another possible miscellaneous cause includes adrenal insufficiency due to ACTH depletion, but this was considered unlikely in our series, given that corticosteroids were routinely administered under the same dosing regimen in all cases. Iatrogenic desmopressin overuse should also be considered as a potential cause of DPH [33]; however, it is unsure whether this was responsible for DPH in our patient #5. Given that SIADH-pattern DPH occurred in patients where desmopressin was not administered (patients #1 and #3), its overdose could not explain the whole picture of their DPHs, and some other pathological processes would have been present as described above. Several other factors are reportedly associated with DPH in patients with pituitary adenoma, such as old age, large tumor size, comorbidity of cardiac, renal, and/or thyroid disease, postoperative cerebrospinal fluid drainage, preoperative hypopituitarism, and female sex. However, none of these factors seem robust [3,4,8,9,10,15,16,33,40,42], and their significance has yet to be determined. We were unable to find any risk factors but extradural gland retraction; however, since our study involved a relatively small number of patients, further study with more patients would be desirable. Even though there is a report where the use of antihypertensive drugs or diuretics was significantly greater in the patients who developed DPH [9], we failed to find any association between patients’ comorbidities (diabetes mellitus, hypertension, and hyperlipidemia) and DPH; however, further study is necessary to reach a conclusion.

The limitations of this study included that the number of patients was relatively small and that evaluations of serum and urine sodium levels were too inconsistent to legitimately analyze those phenomena. Moreover, we did not examine several humoral factors that might also be involved in the water/sodium imbalance in DPH, such as ADH, ANP, brain natriuretic peptide, plasma renin activity, and aldosterone. Further study with a greater number of patients for each tumor pathology with a wider variety of evaluations is thus desirable. Nevertheless, DPH clearly may arise after TSS for NASBTs in the setting of either hypervolemia or hypovolemia due to some pathological processes that were highly likely to have been induced by mechanical stress on the hypothalamo-hypophyseal axis. This appears to be the first study to addresses DPH in NASBTs and, thus, the results are compelling and worth being reported for surgeons engaged in endoscopic TSS.

## 4. Materials and Methods

### 4.1. Patient Selection

We collected data on 66 consecutive patients who had undergone endoscopic TSS between January 2018 and March 2019 at our institution. Exclusion criteria were: (i) pituitary adenoma (*n* = 17); (ii) craniopharyngioma (*n* = 4); (iii) tumor with no parasellar involvement (*n* = 13); (iv) presence of preoperative diabetes insipidus (*n* = 1); and (v) biopsy or partial resection in which the parasellar part remained completely untouched (*n* = 1). Craniopharyngioma was excluded due to a high probability of postoperative diabetes insipidus [43,44]. A total of 30 patients with NASBTs were thus included in the study. All patients provided written informed consent for participation for the present study. All aspects of the study were approved by the institutional review board (The Research Ethics Committee of The University of Tokyo, IRB#2231).

### 4.2. Surgical Procedure

Details of our surgical procedure were as described in the literature [26,45,46]. Briefly, endoscopes 4 mm in diameter (175–180 mm long rigid scopes; Karl Storz Endoscopy Japan, Tokyo, Japan) stabilized with a robotic holding device (Point Setter; Mitaka Kohki, Tokyo, Japan) were used. This allowed two-hand surgery without an endoscopist throughout the procedure. A surgical navigation system (Stealth Station Navigation; Medtronic Japan, Tokyo, Japan) was used in all cases to identify anatomical structures and to help us create an individualized surgical trajectory for each tumor. Basically, the 0-degree scope was used to create the transsphenoidal trajectory and remove tumors located in the midline, and oblique-viewing endoscopes (30- and 70-degree) were mainly used to approach tumors extending to the paramedian retrocarotid space and the anterior third ventricle and to manipulate behind the pituitary gland using an upward view. Manipulations of the pituitary gland was performed only if necessary, and largely fell into (i) extradural retraction of the pituitary gland and (ii) physical contact with the stalk.

### 4.3. Postoperative Management

All patients received postoperative care under a consistent policy. Ringer’s lactate solution or similar isotonic fluid was continuously (50–80 mL/h) used until oral intake was well-tolerated. Patients were allowed to drink water as desired. Fluid balance was strictly monitored throughout the course of postoperative hospitalization. If urine output exceeded > 400 mL/2 h with urine specific gravity < 1.005 in the setting of a negative volume balance, including intraoperative balance, a clinical diagnosis of diabetes insipidus (DI) was made and administration of anti-diuretic hormone (ADH) was started. Unless extreme hypernatremia (>155 mmol/L) developed, oral intake was encouraged without infusion of hypotonic solution. Since postoperative DI is mostly transient, the dose of ADH was adjusted with close monitoring of sodium level and urine output.

Corticosteroids were routinely administered regardless of the presence of pre-operative hypopituitarism, starting with hydrocortisone 100 mg before surgery and every 8 h on POD 0, tapered over five days down to 10–20 mg every morning, and discontinued depending on the level of ACTH and the presence of hypopituitarism. Levels of pituitary hormones were checked before surgery and on POD 7 and further followed in the outpatient clinic if necessary.

Sodium level was routinely checked prior to surgery and at least 3 times postoperatively: on POD 1 (phase 1), POD 2–4 (phase 2), and POD 7–14 (phase 3), and further followed in the outpatient clinic if necessary. A sodium level ≤135 mmol/L was considered hyponatremia [6,10,16], and treatment was initiated if sodium level reached < 130 mmol/L or the patient became symptomatic. The primary management included salt supplementation and/or fluid restriction, individualized based on a combination of the clinical condition and volume status. Volume status is difficult to track precisely, and was thus judged mainly by changes in body weight.

### 4.4. Statistical Analysis

First, baseline characteristics including patient factors (age at time of ETS, sex, preoperative sodium level, and history of hypopituitarism, DI, diabetes mellitus, hyperlipidemia, hypertension, and other significant conditions), tumor factors (pathology, size, location, and history of previous resection and radiotherapy), and surgical manipulations used (dissection at the tumor-gland interface, extradural retraction of the pituitary gland, and physical contact with the pituitary stalk) were summarized. Second, rates of symptomatic and serological DPH were calculated. Third, to evaluate volume status, changes in body weight were calculated as:

$$\frac{\left[\mathrm{baseline}\;\mathrm{body}\;\mathrm{weight}\right]-\;\left[\mathrm{body}\;\mathrm{weight}\;\mathrm{on}\;a\;\mathrm{specific}\;\mathrm{day}\right]}{\left[\mathrm{baseline}\;\mathrm{body}\;\mathrm{weight}\right]}\times \;100\;\left(\%\right).$$

In addition, changes in hematocrit were calculated as:

$$\frac{\left[\mathrm{hematocrit}\;\mathrm{on}\;\mathrm{POD}\;1\right]-\left[\mathrm{hematocrit}\;\mathrm{on}\;a\;\mathrm{specific}\;\mathrm{day}\right]}{\left[\mathrm{hematocrit}\;\mathrm{on}\;\mathrm{POD}\;1\right]}\times \;100\;\left(\%\right),$$

as supportive data to evaluate volume status. Fourth, detailed chronological courses of serum sodium levels were summarized, and changes from preoperative levels were examined with the Wilcoxon signed-rank test. Finally, risk factors associated with symptomatic and serological DPH were examined using Fisher’s exact test (for dichotomous variables) and logistic regression analysis (for continuous variables). When a cell in a 2 × 2 table had a value of zero, Haldane correction was used to calculate the odds ratio [47]. Statistical analyses were performed using JMP Pro version 14.0 (SAS Institute, Cary, NC, USA).

## 5. Conclusions

DPH occurs following TSS not only for pituitary adenomas, but also for NASBTs. Serological and symptomatic DPH occurred in 27% and 13%, respectively on POD 7–12, and were restored within two days with just observation or conservative management. Unlike DPH following TSS for pituitary adenoma, the CSWS pattern was more often observed than the SIADH pattern (Figure 4). Although details of the underlying mechanisms remain unclear, intraoperative extradural compression of the pituitary gland seems to represent a significant risk for DPH. DPH should be recognized as one of the significant postsurgical complications associated with TSS for NASBTs.

## Figures and Tables

**Table 1:** Baseline characteristics.

Variable		Value
Median age, years (range)		47 (19–85)
Median preoperative sodium level, mmol/L (range)		141 (136–148)
Median tumor diameter, mm (range)		31 (15–48)
Male sex, *n* (%)		12 (40)
Comorbidity *, *n* (%)	ACTH-deficiency	0 (0)
	DM	2 (6)
	HL	3 (10)
	HTN	7 (23)
Pathological diagnosis, *n* (%)	Chordoma	15 (50)
	Meningioma	8 (27)
	Chondrosarcoma	5 (17)
	Neurenteric cyst	1 (3)
	Neurinoma	1 (3)
Previous intervention, *n* (%)	Resection	10 (32)
	Radiotherapy	5 (16)
Location *, *n* (%)	Sellar region	10 (33)
	Cavernous sinus	19 (63)
	Suprasellar region	11 (37)
	Dorsum sellae	19 (63)
	Sphenoid sinus	3 (10)
	Petrous apex	12 (40)
	Clivus	22 (73)
	Anterior skull base	4 (13)
	Posterior fossa (intradural)	12 (40)
	Third ventricle	0 (0)

* There is some overlapping. DM = diabetes mellitus; HL = hyperlipidemia; HTN = hypertension.

**Table 2:** The detailed course of serum sodium levels and body weight in patients with serologic hyponatremia.

No.	Age, Sex	Dx	Main Location	Extradural Retraction *	Contact to Stalk	Serum Sodium Level (mmol/L)					Body Weight (kg)			Hematocrit (%)			Symptomatic Hyponatremia
						Pre	Phase 1	Phase 2	Phase 3	Δ **	Pre	Phase 3	Δ% ^†^	Phase 1	Phase 3	Δ% ^‡^	
1	70, F	Mgm	Ptcl	Yes	No	139	138	140	112	−27	54.4	57.3	5.4	38.2	36.1	−5.5	Yes
2	69, F	Cho	Cli	Yes	No	140	143	144	123	−17	51.0	49.2	−3.6	26.2	27.1	+3.4	Yes
3	38, F	Cho	Cli	Yes	No	140	139	140	126	−14	84.3	86.3	2.4	29.9	30.1	+0.3	Yes
4	75, F	Mgm	ITF-Sph	Yes	No	142	141	144	130	−12	43.2	43	−0.5	25.1	31.8	+26.7	Yes
5	58, F	Mgm	TS	No	Yes	141	146	147	130	−11	53.1	57.2	7.7	29.5	25.4	−13.9	No
6	55, F	Mgm	Ptcl	Yes	No	142	139	142	134	−8	60.2	57.7	−4.2	26.3	24.7	−6.1	No
7	59, M	Chs	PA-PF	Yes	No	141	140	136	135	−6	76.7	71.4	−6.9	37.7	43.6	+15.6	No
8	34, M	Cho	DS	Yes	No	137	143	143	134	−3	83.0	77.6	−6.5	38.8	44.1	+13.7	No

* Extradural retraction of the pituitary gland during surgery. ** Changes from pre-operative values. ^†^ Percentage change from pre-operative values. ^‡^ Percentage change from values on postoperative day 1. Cho = chordoma; Chs = chondrosarcoma; Cli = clivus; DS = dorsum sellae; F = female; ITF = infratemporal fossa; M = male; Mgm = meningioma; PA = petrous apex; PF = posterior fossa (intradural); Ptcl = petroclival; Sph = sphenoid sinus; TS = tuberculum sellae.

**Table 3:** Detailed course of sodium levels in patients with no, asymptomatic, and symptomatic DPH.

Clinical Course	Median Serum Sodium Level (Range), mmol/L			
	Pre	Phase 1	Phase 2	Phase 3
No DPH	141	142	143	141
	(136–148)	(136–145)	(136–148)	(136–143)
Asymptomatic DPH	141	142	143	134
	(137–142)	(139–146)	(136–147)	(130–135)
Symptomatic DPH	140	140	142	125
	(139–142)	(138–143)	(140–144)	(112–130)

**Table 4:** Results of statistical analyses for risk factors of DPH.

Variables		Symptomatic DPH		Serologic DPH	
		*p* Value	OR (95%CI)	*p* Value	OR (95%CI)
Age (cont.)		0.098	0.94 (0.86–1.01) *	0.124	0.96 (0.91–1.01) *
Preoperative sodium level (cont.)		0.749	1.09 (0.65–1.82) *	0.624	1.10 (0.74–1.64) *
Tumor diameter (cont.)		0.206	0.93 (0.83–1.04) *	0.413	0.97 (0.89–1.05) *
Male sex		0.120	0.13 (0.01–2.64)	0.419	0.40 (0.07–2.44)
Comorbidity	Diabetes mellitus	1.000	1.09 (0.04–26.67)	1.000	0.48 (0.02–11.14)
	Hyperlipidemia	0.454	2.88 (0.31–26.44)	0.284	3.33 (0.38–28.96)
	Hypertension	1.000	1.68 (0.20–14.09)	0.645	1.50 (0.22–10.36)
Previous intervention	Surgery	0.270	0.15 (0.01–3.07)	0.199	0.17 (0.02–1.64)
	Radiotherapy	0.557	0.35 (0.02–7.41)	1.000	0.49 (0.05–4.94)
Location	Sella	0.095	8.14 (0.72–91.89)	1.000	1.29 (0.24–6.96)
	Cavernous sinus	0.268	6.68 (0.33–136.79)	1.000	0.95 (0.18–5.08)
	Suprasellar region	1.000	0.53 (0.05–5.86)	0.972	0.48 (0.08–2.95)
	Dorsum sellae	0.268	6.68 (0.33–136.79)	0.672	2.08 (0.34–12.72)
	Sphenoid sinus	0.360	4.00 (0.27–58.56)	0.166	7.00 (0.54–91.11)
	Petrous apex	0.632	0.45 (0.04–4.98)	1.000	0.87 (0.16–4.58)
	Clivus	1.000	1.11 (0.10–12.47)	1.000	1.13 (0.18–7.19)
	Anterior skull base	1.000	0.56 (0.03–12.24)	1.000	0.90 (0.08–10.21)
	Posterior fossa (intradural)	0.130	0.13 (0.01–2.64)	0.433	0.40 (0.07–2.44)
Extradural retraction of the gland		0.100	12.13 (0.59–248.50)	0.035 **	12.25 (1.27–118.36)
Physical contact with the stalk		1.000	0.43 (0.02–9.34)	1.000	0.64 (0.06–6.80)

cont. = continuous variable; OR = odds ratio; * Unit odds ratio. ** A *p* < 0.05 is considered significant.
